# Morphogenetic penicillin-binding proteins control virulence-associated type III secretion systems in *Salmonella*

**DOI:** 10.1128/iai.00555-24

**Published:** 2024-12-31

**Authors:** Sónia Castanheira, Sara Torronteras, Juan J. Cestero, Francisco García-del Portillo

**Affiliations:** 1 Laboratory of Intracellular Bacterial Pathogens, National Centre for Biotechnology (CNB-CSIC) 54447 , Madrid, Spain; University of California Davis, Davis, California, USA

**Keywords:** *Salmonella*, PBP2, PBP3, PBP2_SAL_, PBP3_SAL_, type III secretion system

## Abstract

Type III protein secretion systems (T3SSs) function as multiprotein devices that span the envelope of Gram-negative bacteria using the peptidoglycan (PG) layer as scaffold. This spatial arrangement explains why modifications in PG structure can alter T3SS activity. In *Salmonella,* incorporation of non-canonical D-amino acids in the PG was shown to decrease the activity of the T3SS encoded by the pathogenicity island-1 (SPI-1) without affecting other T3SS, like the flagellum apparatus. Enigmatically, following invasion of host cell *Salmonella enterica* serovar Typhimurium modifies PG synthesis by upregulating two pathogen-specific enzymes, the penicillin-binding proteins PBP2_SAL_ and PBP3_SAL_, with roles in cell elongation and division, respectively. In the mouse typhoid model, the amount of PBP2_SAL_ and PBP3_SAL_ produced by the pathogen exceeds by large those of the canonical enzymes PBP2 and PBP3. This change responds to acidity and high osmolarity, the same cues that intra-phagosomal *S*. Typhimurium perceives to switch the SPI-1 T3SS by that encoded in SPI-2. Using isogenic mutants lacking each of the four morphogenetic PBPs, we tested whether their activities and those of the T3SS encoded by SPI-1 and SPI-2, are interconnected. Our data show that PBP2 is required for proper function of SPI-1 T3SS but dispensable for motility, whereas the lack of any of the morphogenetic PBPs increases SPI-2 T3SS activity. The positive control exerted by PBP2 on SPI-1 takes place via the SPI-1-specific regulators HilA and InvF. To our knowledge, these findings provide the first evidence linking morphogenetic enzymes that synthesize PG with T3SS associated to virulence.

## INTRODUCTION

The peptidoglycan (PG) is the component of the bacterial cell envelope that defines cell shape and ensures cell integrity against external insults and turgor (1–3). The PG structure consists of glycan chains cross-linked by short peptides built by an elaborated biosynthetic pathway in which many cytosolic, integral, and membrane-associated enzymes participate (1, 2). A hallmark of the PG metabolism is the intermediate molecule named lipid II, which acts as a building block flipped from the cytosolic leaflet of the inner membrane to the periplasm (4). Lipid II is a lipid-bound disaccharide bearing a peptide stem, consisting of undecaprenyl-P-P-N-acetyl-muramyl-(stem-peptide)-N-acetyl-glucosamine. The stem peptide of lipid II terminates in D-Ala–D-Ala, a bond that is cleaved by two different classes of enzymes. The first are D,D-transpeptidases that cross-link stem peptides of adjacent glycan chains, incorporating in this manner new material to nascent PG. The second class are D,D-carboxypeptidases that cleave the D-Ala–D-Ala bond and as consequence, decreases the degree of cross-linking (5). The recognition of the D-Ala–D-Ala motif by these two groups of enzymes is the basis of their capacity to bind beta-lactam antibiotics and why they were coined as penicillin-binding proteins (PBPs) (6). Early studies denoting morphological changes in cells exposed to distinct beta-lactam antibiotics identified a subgroup of PBPs involved in PG synthesis, which control cell elongation and cell division (7). These morphogenetic PBPs are monofunctional transpeptidases (TPases) termed class B PBPs (bPBPs) that interact with cognate glycosyl-transferases (GTases) to incorporate lipid II to nascent PG. In *Escherichia coli*, these TPase–GTase pairs are PBP2/RodA and PBP3(FtsI)/FtsW, which regulate cell elongation and division, respectively (1, 3). Besides morphogenetic PBPs, a second group of enzymes known as bifunctional class A PBPs (aPBPs) catalyze both TPase and GTase reactions (8).

As giant covalently bound meshwork covering the entire bacterial surface, the PG plays an important structural role in which many multiprotein devices assemble. One of these macromolecular structures is the flagellar apparatus, which is built as a basal body spanning the envelope followed by a conduit in which the flagellin is secreted unprocessed. The flagellum apparatus is an example of type III protein secretion system (T3SS) (9), which has been intensively studied in the context of motility in environmental and pathogenic bacteria, the structure of its components, and the dynamics of the assembly process (10). Among the proteins of the flagellar basal body, some interact with the PG, providing the required stability to the machinery. Equally relevant is the role played by enzymes that degrade locally the PG to facilitate assembly of the apparatus. Some of the best characterized flagellum-specific PG hydrolases are FlgJ of *Salmonella enterica* serovar Typhimurium (*S*. Typhimurium) displaying beta-N-acetyl-glycosaminidase activity (11), and the lytic transglycosylase SltF of *Rhodobacter sphaeroides* (12). PG hydrolases also contribute to the secretion of virulence factors. In *S*. Typhi, the secretion of typhoid toxin depends on the concerted action of the L,D-transpeptidase YcbB and the hydrolase TstA with N-acetyl-muramidase activity (13). TstA was proposed to recognize YcbB-edited PG at the cell poles to spatially define a site in which typhoid toxin could be secreted (14, 15). Specialized hydrolases with lytic transglycosylase or muramidase activities have also been reported for other T3SS and T4SS (16). In enteropathogenic *E. coli*, the lytic transglycosylase EtgA interacts with EscI, an inner rod protein of the T3SS basal body and is required for efficient T3SS secretion (17, 18). PG-degradative activities were also reported for IpgF and IagB, involved in virulence-associated T3SS of *Shigella flexneri* and *S. enterica*, respectively (16); HpaH in a T3SS of *Xanthomonas campestris* (19); VirB1 in T4SS of *Agrobacterium tumefaciens* (20); and VirB1 homologs of *Brucella suis* and *Streptococcus suis* (21, 22).

Studies in *S*. Typhimurium demonstrated that alterations in PG structure caused by *in vivo* incorporation of non-canonical D-amino acids compromises the activity of the invasion-associated T3SS encoded by the *Salmonella*-pathogenicity island-1 (SPI-1) (23). Such effect is associated to a reduced number of needle complexes, which indicates failure in the capacity of the bacterium to efficiently assemble the SPI-1 T3SS apparatus. Our recent data also favor changes in the intracellular niche regarding the PG biosynthetic machinery. These include the upregulation of the pathogen-specific D,L-endopeptidase EcgA, which cleaves the D-glutamic acid (D-Glu)-*meso*-diaminopimelic acid (*meso*-Dap) bond in stem peptides of the PG (24), and the morphogenetic PG synthases PBP2_SAL_ and PBP3_SAL_ (25, 26). Noteworthy, expression of these pathogen-specific enzymes involved in PG metabolism is controlled by the virulence-associated transcriptional regulators PhoP and OmpR (24, 27). These two regulators are also responsible for switching the invasion-associated SPI-1 T3SS to the secretion system encoded by SPI-2, essential for *S*. Typhimurium to survive and proliferate within the phagosomal compartment of host cells (28, 29).

In this study, we tested the hypothesis of a putative functional link between activities of enzymes involved in PG synthesis and those of the SPI-1 and SPI-2 T3SS. The data support an involvement of morphogenetic PBPs in modulating activities of the SPI-1- and SPI-2-encoded T3SS associated to virulence.

## RESULTS

### PBP2 is required for activity of the invasion-associated SPI-1 T3SS

The presence in *S*. Typhimurium of PBP2_SAL_ and PBP3_SAL_, the two pathogen-specific morphogenetic PBPs that can replace PBP2 and PBP3 in infection conditions, allowed us to generate null mutants lacking each of the four PBPs (25, 26, 30, 31). These *S*. Typhimurium isogenic mutants were tested for SPI-1 T3SS activity by monitoring the profile of secreted proteins following overnight growth in nutrient-rich LB medium, pH 5.8. This condition is permissive for mutants lacking the canonical PBP2 or PBP3, which depend on acidic pH for growth (25, 31). SPI-2 T3SS activity, known to be prominent in response to acidity (32), was monitored in acidified nutrient-limited minimal medium phosphate–carbon–nitrogen (PCN) pH 4.6, in which production of the pathogen-specific enzymes PBP2_SAL_ and PBP3_SAL_ is up-regulated (27).

**Fig 1:**
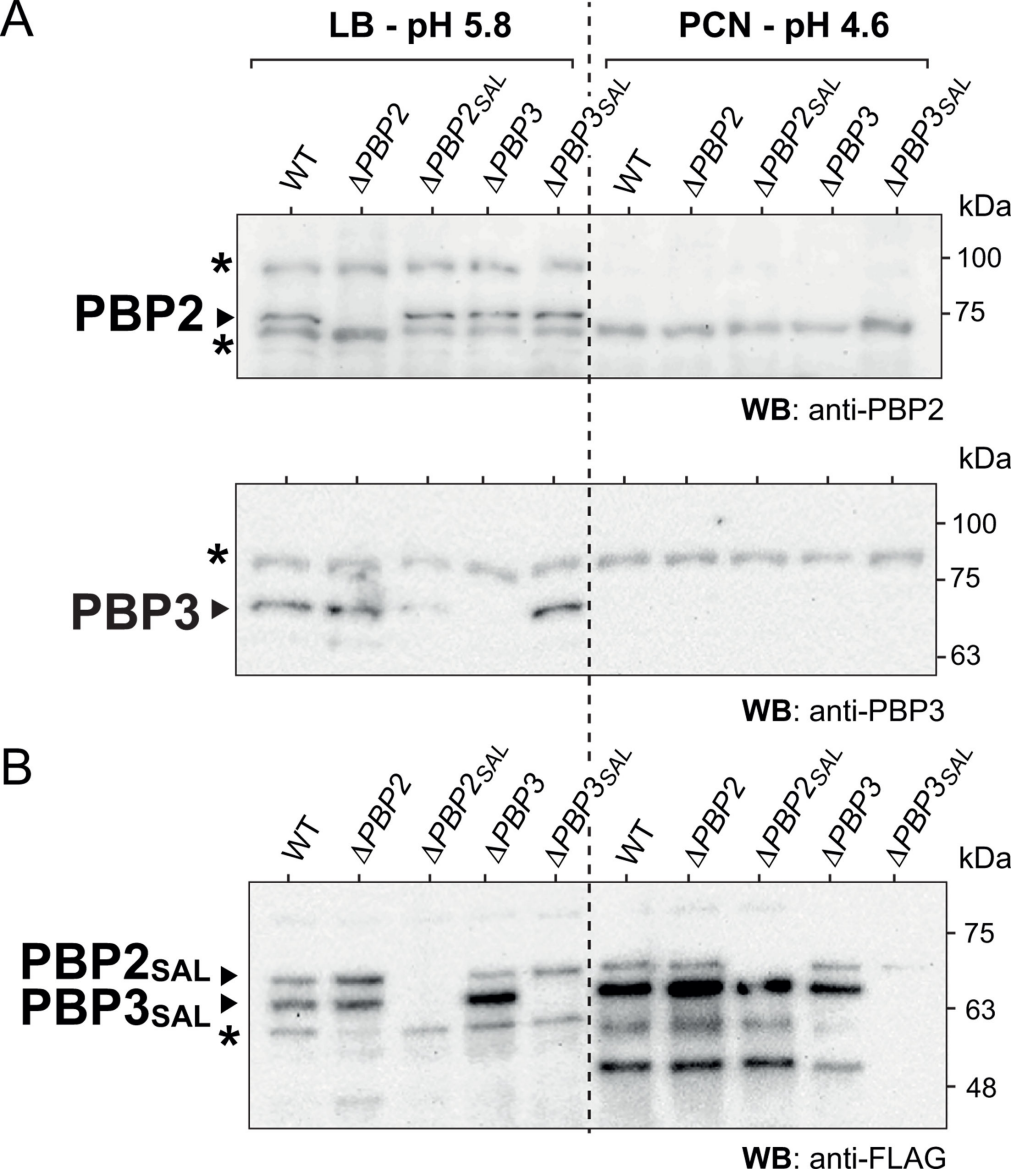
Levels of morphogenetic peptidoglycan (PG) synthases in isogenic *S*. Typhimurium mutants lacking canonical (PBP2, PBP3) or pathogen-specific (PBP2_SAL_, PBP3_SAL_) enzymes involved in cell elongation or cell division. Shown are Western blots of total protein extracts of the indicated isogenic strains grown to stationary phase in LB pH 5.8 or nutrient-limited PCN 150 mM NaCl 0.4% glucose pH 4.6 media. (**A**) Levels of the canonical enzymes detected with anti-PBP2 or anti-PBP3 antibodies; (**B**) Levels of tagged versions of PBP2_SAL_/PBP3_SAL_ detected with anti-FLAG antibody. Asterisks denote non-specific bands. Position and size (in kDa) of the molecular weight protein markers are indicated. Note the downregulation of PBP2 and PBP3 when bacteria grow in SPI-2-inducing conditions—nutrient-limited PCN pH 4.6 medium—consistent with our previous reports (27). Data are representative of two independent biological replicates.

Prior to testing T3SS activities, we monitored in LB pH 5.8 and PCN pH 4.6 conditions the relative amount compared with wild-type cells of morphogenetic PBPs produced by the four isogenic mutants Δ*PBP2*, Δ*PBP3*, Δ*PBP2_SAL_*, and Δ*PBP3_SAL_*. This experiment corroborated our previous findings (27), including downregulation of PBP2 and PBP3 in the SPI-2 T3SS-inducing condition (PCN, pH 4.6, 150 mM NaCl, 0.4% glucose) (Fig. 1A) and an apparent absence of PBP3_SAL_ in the Δ*PBP2_SAL_* mutant growing in LB pH 5.8 (Fig. 1B).

**Fig 2:**
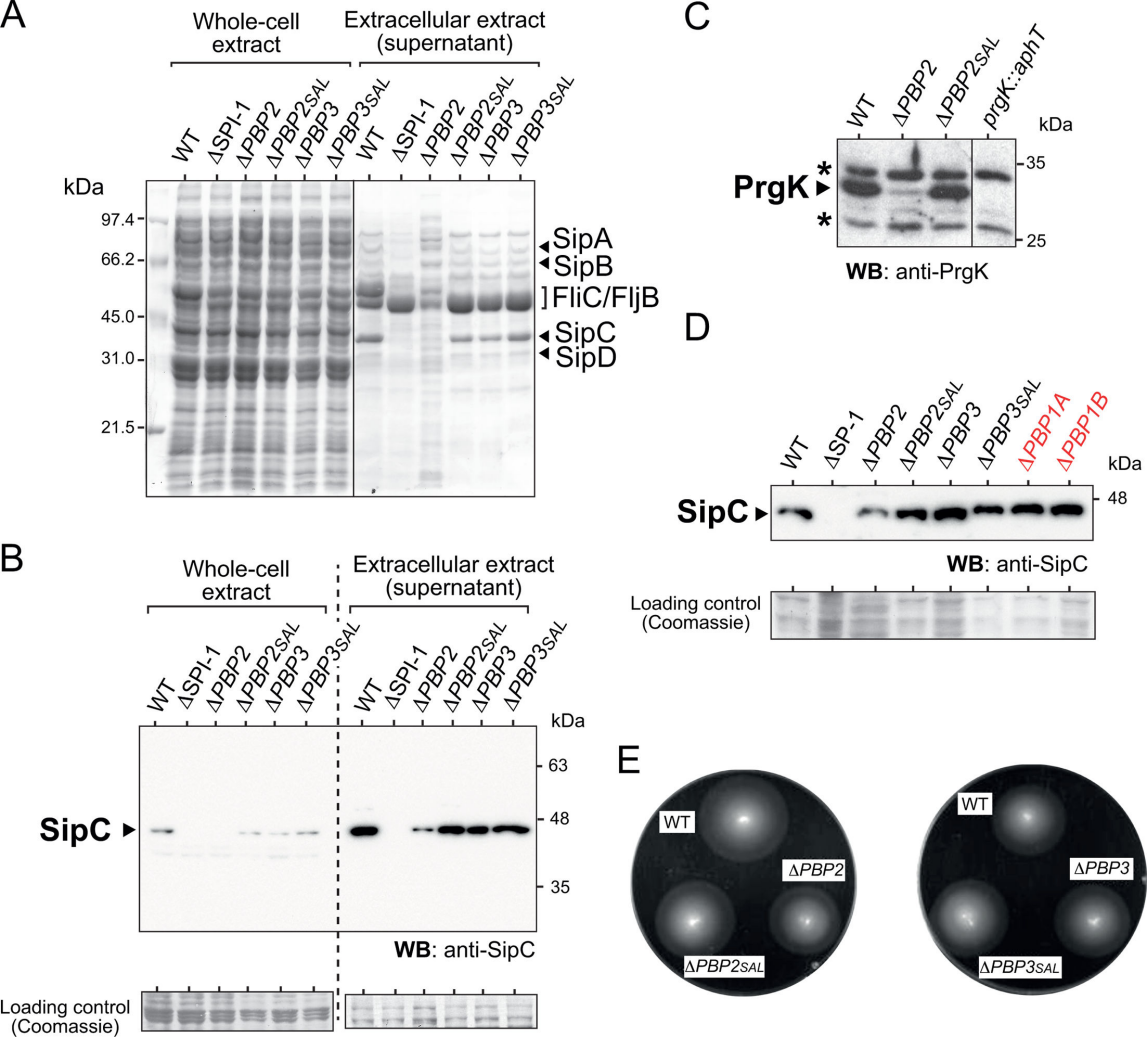
The morphogenetic PBP2 is required for SPI-1 T3SS activity. (**A**) Coomassie staining of whole cell-associated proteins and secreted proteins of the indicated strains grown in LB pH 5.8 to stationary phase. The position of the SPI-1 proteins SipA, SipB, SipC, and SipD is indicated, as well as flagellins FliC and FljB. Positions and size (in kDa) of molecular weight markers are shown. (**B**) Levels of SipC detected by Western blotting in whole cell-associated and secreted protein extracts of the indicated isogenic mutants. The volumes loaded of whole cell and supernatant extracts in panels (**A**) and (**B**) correspond to 2.75 × 10^7^ and 2.75 × 10^9^ bacteria, respectively. Coomassie staining of the PVDF membrane used in the Western blot is shown as loading control; (**C**) Levels of the SPI-1 structural protein PrgK in mutants lacking PBP2 or PBP2_SAL_ grown overnight in LB pH 5.8. A null *prgK* mutant was run in parallel as negative control. Asterisks denote non-specific bands; (**D**) Secretion of SipC is not altered in the absence of the major PG synthases PBP1A or PBP1B. The Coomassie staining of the same samples run on a gel is shown as loading control; (**E**) Motility assay of *S*. Typhimurium mutants lacking morphogenetic PBPs. Shown are the motility halos observed after 6 h of growth in semi-solid motility medium pH 4.6 (see Materials and Methods). Data are representative of two biological replicates.

We next determined the capacity of these mutants to secrete proteins using SPI-1 T3SS, including a ΔSPI-1 null mutant lacking this pathogenicity island as negative control (33). Figure 2A shows that among the four morphogenetic mutants, only Δ*PBP2* has an altered protein profile in the extracellular (supernatant) fraction, indicative of cell envelope alterations impacting protein secretion. To define the extent at which this alteration was affecting SPI-1 T3SS activity, we determined the levels of the SPI-1 translocon protein SipC and the structural protein PrgK, with the latter being a component of the needle complex basal body (34). Compared with the rest of the strains, SipC and PrgK were detected at lower levels in the extracellular and whole-cell extracts of the Δ*PBP2* mutant (Fig. 2B and C). Isogenic mutants lacking PBP1A or PBP1B, the bifunctional aPBPs not directly implicated in morphogenesis (8), did not show, however, alterations in their capacity to secrete SipC (Fig. 2D). Interestingly, no effect on motility was observed for any of the four morphogenetic mutants tested (Fig. 2E). Taken together, these findings support a requirement of the morphogenetic PG synthase PBP2 for activity of SPI-1 T3SS.

### PBP2 facilitates *S.* Typhimurium entry into eukaryotic cells

**Fig 3:**
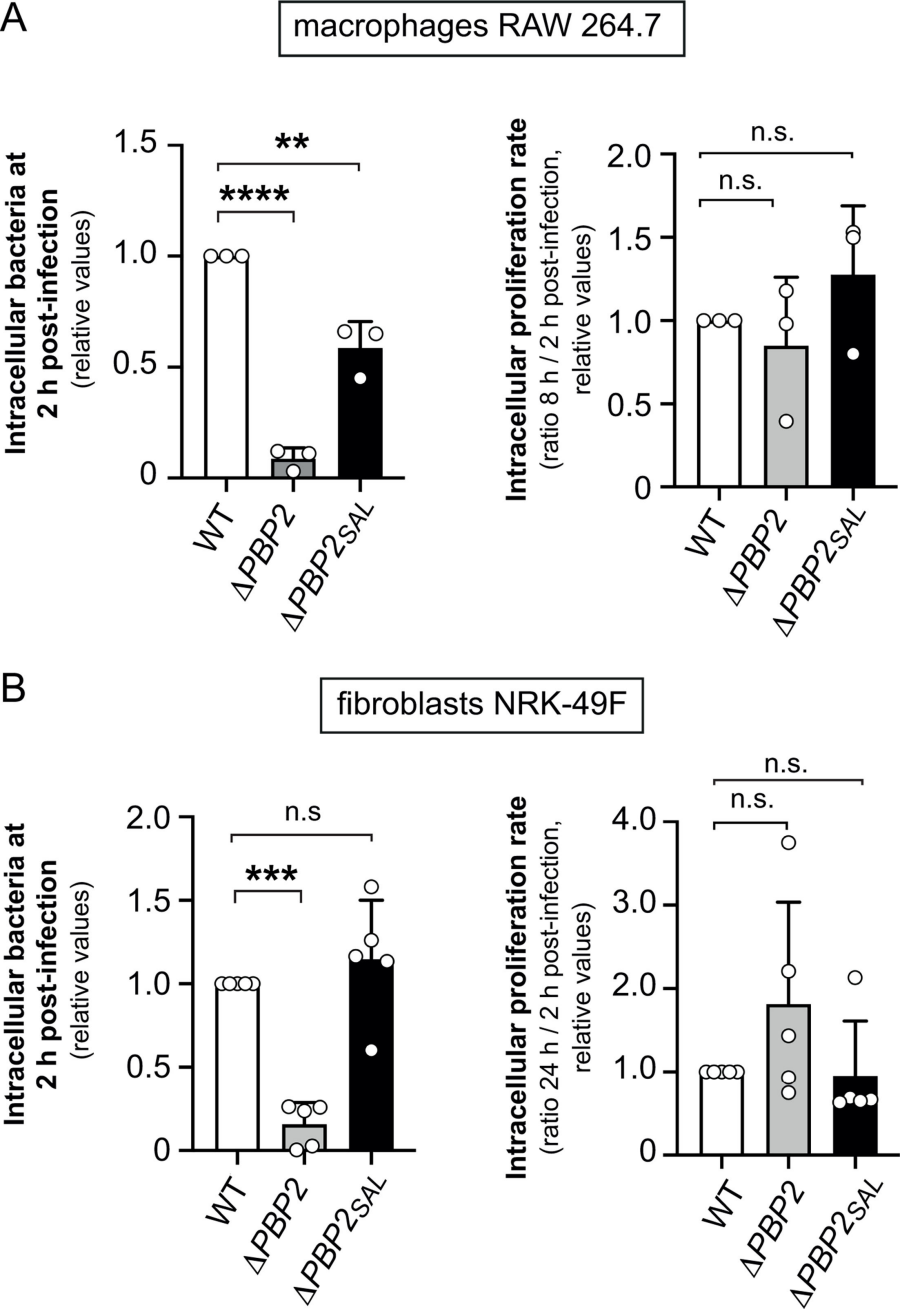
The morphogenetic PBP2 modulates entry of *S*. Typhimurium into phagocytic and non-phagocytic cells. (**A**) Invasion and intracellular proliferation rates of macrophages RAW 264.7 by *S*. Typhimurium mutants lacking PBP2 or PBP2_SAL_. Shown are the mean and standard deviations of three independent biological replicates relative to the values of wild-type cells; (**B**) same as panel (**A**) for NRK-49F fibroblasts. In this infection model, data are from five independent biological replicates. Note that the absence of PBP2 results in significant decrease of bacterial entry into both phagocytic and non-phagocytic cells, whereas that of PBP2_SAL_ also slightly affects uptake by macrophages. Bacteria were grown overnight in non-shaking conditions at 37°C in LB pH 5.8 medium. Statistical analysis was performed as one-way ANOVA with multiple comparison and Tukey’s post-test. n.s., not significant; **, *P* ≤ 0.01; ***, *P* ≤ 0.001; ****, *P* ≤ 0.0001.

As the lack of PBP2 correlates to decreased SPI-1 T3SS activity (Fig. 2), we reasoned that the absence of this PG synthase could impact *S*. Typhimurium entry into eukaryotic cells. To test this, we used mouse RAW 264.7 macrophages pre-activated with γ-interferon and rat NRK-49F fibroblasts, with the latter being a non-phagocytic cell type in which we have extensively characterized the control of growth rate in intra-phagosomal *S*. Typhimurium (35). The number of intracellular bacteria in macrophages at early infection times was in an average ~10-fold lower in the Δ*PBP2* strain compared with wild-type cells (Fig. 3A). For the Δ*PBP2_SAL_* strain, which produces PBP2 under this condition (Fig. 1A), a slight decrease in uptake of ~twofold compared with wild-type cells was also observed (Fig. 3A). Interestingly, survival rates inside the macrophage up to 8 h post-infection were indistinguishable regardless of the presence/absence of PBP2 or PBP2_SAL_ (Fig. 3A). In the non-phagocytic infection model of fibroblasts, the Δ*PBP2* mutant exhibited a ~sixfold less invasion rate compared with wild-type or Δ*PBP2_SAL_* cells (Fig. 3B). No significant differences were observed in the intracellular/survival rate in the strains lacking PBP2 or PBP2_SAL_ (Fig. 3B). Overall, these data inferred that the lack of PBP2 in *S*. Typhimurium affecting SPI-1 activity results in reduced uptake by phagocytic cells and invasion of non-phagocytic cells.

### PBP2 modulates SPI-1 T3SS activity via the transcriptional regulators HilA and InvF

**Fig 4:**
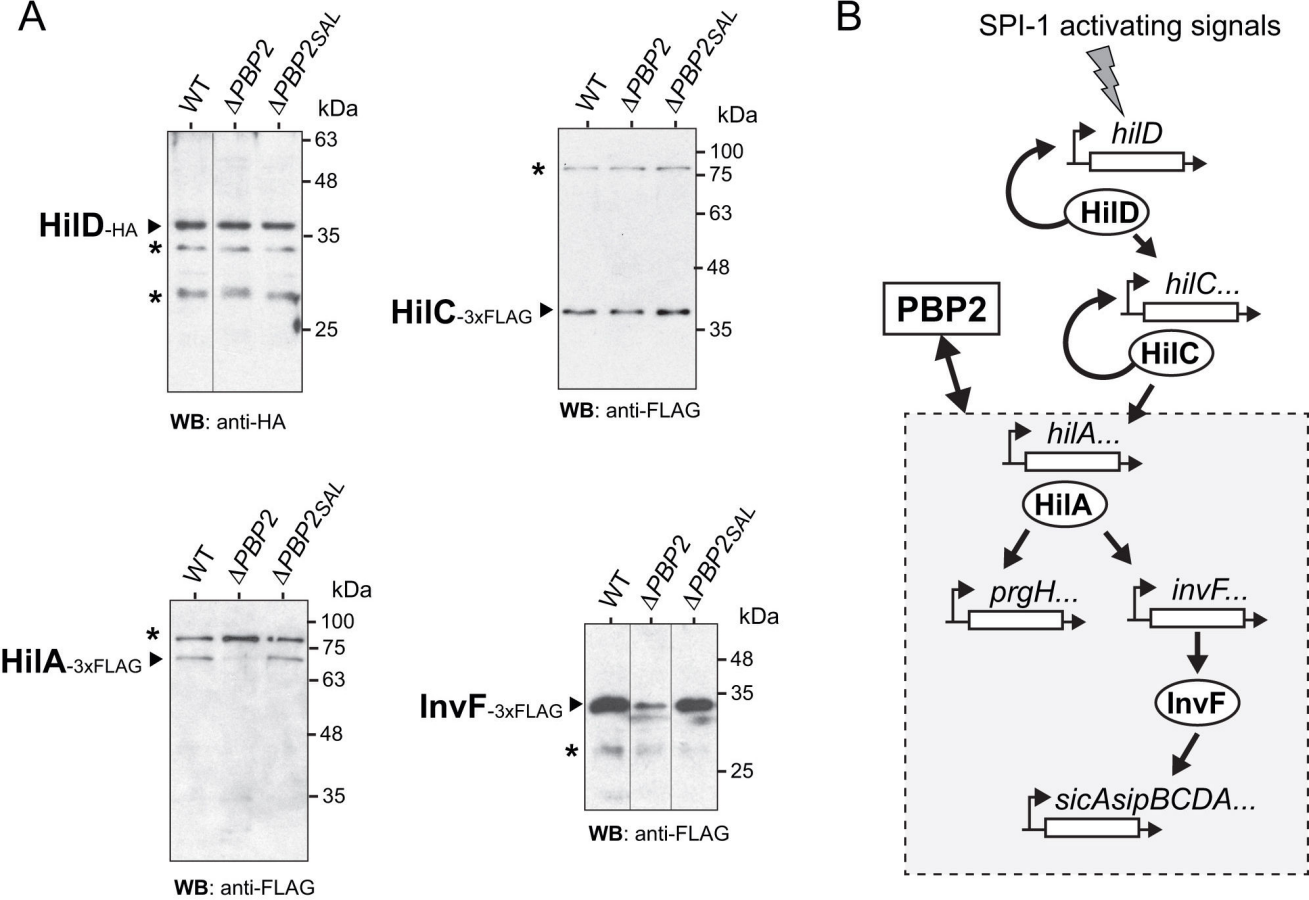
The lack of PBP2 in *S*.Typhimurium results in lower levels of the SPI-1 transcriptional regulators HilA and InvF. (**A**) Relative levels of HilD, HilC, InvF, and HilA detected by Western blotting in whole cell extracts of the indicated isogenic strains lacking either PBP2 or PBP2_SAL_ grown to stationary phase in LB pH 5.8 medium. The strains bear either HA- or 3xFLAG-tagged alleles of *hilD, hilC*, *invF* and *hilA* in their respective native chromosomal locations. Data are representative of two biological replicates; (**B**) Scheme depicting the regulatory cascade that activates SP-1 gene expression and genes encoding SPI-1 effectors (36). RtsA, a SPI-1 regulator that like HilA acts downstream of HilC (38), is not included as it was not analyzed. The section of the cascade sensitive to the activity of PBP2 is shadowed in gray.

SPI-1 expression is controlled by an intricated network of transcriptional regulators acting hierarchically (36). The structural protein PrgK, encoded by a gene mapping in SPI-1, is produced at lower levels in the absence of PBP2 (Fig. 2B). This finding led us to hypothesize that the structural changes in PG associated with a deficiency in PBP2 could be sensed by dedicated SPI-1 transcriptional regulators causing a global effect on the SPI-1 T3SS. To test this, we determined levels of the upstream regulators HilD and HilC, and those of HilA, InvF, these two latter downstream activators of SPI-1 genes and others mapping elsewhere in the genome encoding effector proteins translocated by SPI-1 T3SS (36, 37). Western blotting revealed that, in contrast to HilD and HilC, which are produced at constant levels regardless of the presence/absence of PBP2 or PBP2_SAL_, the levels of HilA and InvF decrease significantly in the Δ*PBP2* mutant (Fig. 4A). These data support the requirement of a functional PBP2 for progression of the regulatory cascade that controls SPI-1 gene expression at the level of the downstream regulators HilA and InvF (Fig. 4B).

### The activity of SPI-2 T3SS depends on a balanced ratio of morphogenetic PBPs

**Fig 5:**
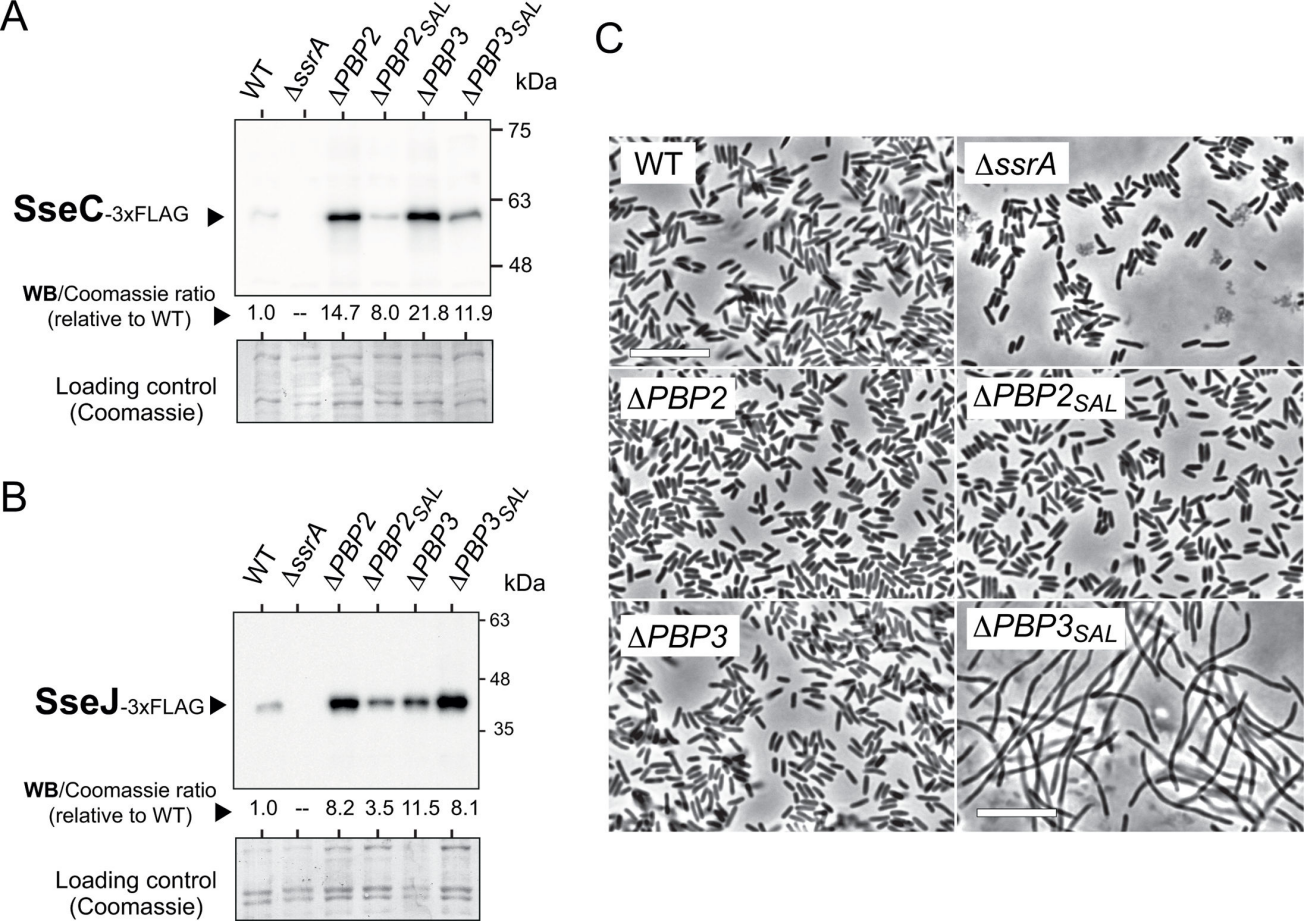
A balanced ratio of morphogenetic PBPs defines expression level of T3SS SPI-2. (**A**) Levels of the translocon protein SseC of T3SS SPI-2 detected in whole cell-associated extracts from the indicated series of isogenic mutants lacking morphogenetic PBPs. As loading control, the Coomassie staining of the PVDF membrane is shown below. Bacteria were grown in nutrient-limited medium PCN 150 mM NaCl, 0.4% glucose, pH 4.6; (**B**) Levels of the SPI-2 effector SseJ in the same *in vitro* conditions as in panel (**A**). The ratio of densitometry values obtained in the Western and Coomassie staining is indicated in both panels; (**C**) Microscopy images of the indicated isogenic mutants grown in the SPI-2 inducing conditions, PCN pH 4.6 with 150 mM NaCl and 0.4% glucose. Note that the lack of PBP3_SAL_ under this condition causes a severe blockage of cell division. Bar, 10 µm. As negative control for SPI-2 activity, all assays included a mutant lacking the master SPI-2 regulator SsrA. Data are representative from a minimum of two independent biological replicates.

*S*. Typhimurium upregulates PBP2_SAL_ and PBP3_SAL_ production in response to signals like acidity and high osmolarity (25, 27). PBP2_SAL_ and PBP3_SAL_ are also expressed at high levels *in vivo* by bacteria located inside phagosomes of cultured mammalian cells or target organs of infected mice (26). Since these conditions are reminiscent of those inducing SPI-2 T3SS, we hypothesized that changes in PG structure linked to the activity of PBP2_SAL_ and PBP3_SAL_ could favor activity of this T3SS. The isogenic *S*. Typhimurium mutants lacking morphogenetic PBPs were grown in acidified nutrient-poor minimal medium PCN pH 4.6 with 150 mM NaCl and 0.4% glucose and tested for the levels of SseC, a translocon protein of the SPI-2 injectosome (39, 40). As we previously reported, under these nutrient-limiting conditions resembling those of the phagosomal compartment, *S*. Typhimurium shows limited capacity to grow with increments of only about two- to threefold of the initial OD values (27). Surprisingly, this reduced growth condition is the most suitable to reproduce *in vitro* the replacement of PBP2/PBP3 by PBP2_SAL_/PBP3_SAL_ that occurs in the pathogen when colonizing animal tissues (26, 27). The Δ*PBP2* and Δ*PBP3* mutants showed increased levels of SseC in the whole cell-associated extract (Fig. 5A), suggesting at a first instance that the exclusive presence of PBP2_SAL_ and PBP3_SAL_ controlling PG synthesis and morphogenesis could exacerbate the expression of the SPI-2 T3SS. However, although at a lower extent, the Δ*PBP2_SAL_* and Δ*PBP3_SAL_* mutants also produced higher levels of SseC compared with wild-type cells (Fig. 5A). This result led us to hypothesize that the balanced ratio of the four morphogenetic PBPs could signal the cell to establish a proper level of activity for SPI-2 T3SS. Consistent with this model, the relative levels of the SPI-2 effector SseJ produced by these mutants followed similar trends compared with SseC, with notorious increases in the Δ*PBP2* and Δ*PBP3* mutants and relatively lower increases in the Δ*PBP2_SAL_* and Δ*PBP3_SAL_* cells (Fig. 5B). It is also relevant to note that, in agreement with our previous studies (25), the Δ*PBP3_SAL_* mutant shows a defect in cell division in these infection-mimicking conditions (Fig. 5C), which may also contribute to the altered expression of the SPI-2 T3SS. Despite several attempts to detect secretion of SseJ to the extracellular milieu, we did not succeed in detecting this effector in reasonable amounts in the supernatant fraction, which precluded further analyses regarding the secretion activity of SPI-2 T3SS in *in vitro* conditions. These findings relative to SseJ secretion are consistent with the lack of reports showing active secretion of SPI-2 effectors in *in vitro* conditions, and studies in pathogenic *E. coli* support fine regulation of effector secretion that is only prominent upon host cell contact or mutations in regulatory proteins (41, 42). We conclude from our data that the expression of the SPI-2 T3SS in *S*. Typhimurium is modulated by a balanced ratio between canonical and pathogen-specific morphogenetic PBPs.

## DISCUSSION

Virulence-associated type III secretion systems (T3SSs) have been intensively studied, with reports providing detailed structural data of protein–protein interactions that take place during injectosome assembly (34, 43). Contrasting with this bulk of information, the knowledge is significantly scarce concerning the association of this macromolecular device with the peptidoglycan (PG) layer. Apart from a few PG hydrolases proposed to facilitate penetration of the PG by the rod region of T3SS or T4SS apparatuses (16), not much is known about the mechanism by which the basal body of the multiprotein device is tethered to the PG scaffold. To date, the best characterized protein–PG interaction is that of the flagellar stator MotB with PG via a C-terminal region that is conserved in other PG-associated proteins (44). InvH, a component of the *S*. Typhimurium SPI-1 T3SS, was also shown by *in vivo* cross-linking assays to be associated with the PG (23). Similarly, a report showed that HrpB1 of *Xanthomonas* spp. binds to PG and to distinct T3SS proteins (45).

In this study, we hypothesized that *S*. Typhimurium enzymes involved in PG synthesis could control the activity of the invasion-associated SPI-1 T3SS. This new concept is based on our previous findings, which revealed that changes in the PG structure following *in vivo* incorporation of non-canonical D-amino acids reduce the number of needle complexes in *S*. Typhimurium (23). Surprisingly, this change does not affect assembly or function of the flagellar apparatus, which supports unique structural determinants in the PG recognized separately during assembly of the flagellum or the SPI-1 T3SS. Moreover, we reasoned that the presence in *S*. Typhimurium of pathogen-specific proteins involved in the last steps of PG synthesis, modification, and morphogenesis (24, 25, 31, 46) could generate such hypothetical PG structural determinants guiding the assembly or location of virulence-associated T3SS. Our results point to PBP2 as the most important PG synthase of the bPBP class that provides envelope stability and ensures proper activity of the SPI-1 T3SS. Interestingly, PBP2 has as partner the glycosyltransferase RodA in the multiprotein complex that promotes cell elongation, known as elongasome (47). Our results raise the possibility of the elongasome being co-opted in *S*. Typhimurium to activate virulence-associated functions, for example, by regulating the location and the number of T3SS complexes to be assembled on the bacterial surface. This idea reconciliates with an early study in *S*. Typhimurium that showed a requirement of the actin-homolog MreC, a key component of the cytoskeleton scaffold that guides elongasome movement, in the expression of the SPI-1 T3SS and flagellar systems (48). However, our data differ in part from this study since the lack of PBP2 has no effect on flagella synthesis or motility but results in altered SPI-1 T3SS activity (Fig. 2A through D). A plausible explanation to this discrepancy is that we used acidic pH in our assays, condition in which the Δ*PBP2* mutant is fully viable and produces the alternative PG synthase PBP2_SAL_ (31). PBP2_SAL_ could therefore ensure some degree of cell elongation sufficient for assembly of the flagellar apparatus. Overall, our data favor the idea of assembly of the invasion-associated SPI-1 T3SS requiring some structural change(s) in the PG layer catalyzed by PBP2, a monofunctional bPBP with D,D-transpeptidase activity.

Our study also shed light into the mechanism linking PBP2 activity and that of SPI-1 T3SS. The data support a model with alteration of the envelope architecture in the absence of PBP2, resulting in lower expression of the regulators HilA and InvF. A study by Palmer and Slauch in *S*. Typhimurium reported that envelope stress caused by the lack of BamB, a protein essential in the β-barrel assembly machinery, leads to repression of SPI-1 expression (49). These authors linked lower *hilA* transcription to activation of the RcsCDB signaling cascade, which responds to envelope damage caused by agents that perturb PG structure or synthesis like lysozyme or beta-lactam antibiotics (50) Treatment of *E. coli* cells with mecillinam, a beta-lactam that blocks specifically PBP2 (51), induces the RcsCDB phosphorelay (52). Based on these findings, it could be speculated that the negative effects we see in the SPI-1 T3SS in *S*. Typhimurium lacking PBP2 could be RcsCDB-dependent. Nonetheless, our data are not entirely consistent with this study since, upon activation, the RcsCDB system represses flagellar synthesis in *E. coli* and *S*. Typhimurium (50, 53), and we did not detect a motility phenotype in cells lacking PBP2. We therefore conclude that the RcsCDB system could be activated in the *S*. Typhimurium Δ*PBP2* mutant although at a much lower extent than when adding beta-lactam antibiotics. It is also important to note that the alternative pathogen-specific D,D-transpeptidase PBP2_SAL_, although produced in the acidic conditions used in our assays, is unable to restore the invasion rate of the Δ*PBP2* mutant to the level of wild-type cells neither in phagocytic nor in non-phagocytic cells. This result suggests that the biosynthetic activity of PBP2 that facilitates SPI-1 T3SS assembly cannot be accomplished by PBP2_SAL_. On the other hand, the absence of PBP2_SAL_ reduces by ~twofold the uptake of *S*. Typhimurium by macrophages, which indicates that this morphogenetic PBP can also introduce some structural changes in the PG that may affect recognition of the pathogen by the phagocyte. Despite this, our study demonstrates a predominant role of PBP2 in the initial *S*. Typhimurium–host cell interaction, reinforced by the fact that none of the two major bifunctional PG synthases, PBP1A or PBP1B, is essential for SPI-1 T3SS activity. Globally, these findings point to HilA and InvF as putative sensors of the integrity and functional status of the envelope, a phenomenon to which to our knowledge there is no precedent. Although not tested here, it will be also of interest to monitor levels of RtsA in the absence of PBP2, considering tha this regulator also acts at the stage that HilA does in the cascade controlling SPI-1 (38).

With respect to SPI-2 T3SS, our data fit in a model in which a balanced ratio of canonical and pathogen-specific PG synthases could be necessary to establish the threshold activity of this important T3SS, used by *S*. Typhimurium inside host cells (28). Despite the fact that our initial data in the minimal PCN pH 4.6 medium indicated that the Δ*PBP2_SAL_* and Δ*PBP3_SAL_* mutants produce levels of PBP2 and PBP3 that are undetectable by Western blot, the increased levels of SseC and SseJ exhibited by these two mutants do not rule out the production of minor amounts of the canonical morphogenetic PBPs under this growth condition. On the other hand, the stronger phenotype showed by Δ*PBP2* or Δ*PBP3* cells regarding expression levels of SseC and SseJ agrees with the upregulation of PBP2_SAL_ and PBP3_SAL_ by intracellular *S*. Typhimurium (26). With the available data, it remains however unknown whether, similarly to SPI-1 T3SS, the mechanism by which morphogenetic PBPs control SPI-2 T3SS involves changes in the SPI-2 specific two-component system SsrA/SsrB, a hypothesis that deserves to be investigated. It is also intriguing that the expression of PBP2_SAL_ and PBP3_SAL_ is fully dependent on EnvZ/OmpR (27), a two-component system that plays a key role in SPI-2 activation (54). In our opinion, this study paves the way to investigate more in depth the probable convergent evolution existing between activities acquired by *S*. Typhimurium involving virulence-associated T3SS and PG structural patterns linked to morphogenetic PG synthases.

## MATERIALS AND METHODS

### Bacterial strains and growth conditions

The *S*. Typhimurium strains used in this study derive from wild-type strain SV5015 (55) and are listed in Table 1. Bacteria were grown in nutrient-rich lysogenic broth (LB), composed of 10 g/L tryptone, 5 g/L yeast extract, and 5 g/L sodium chloride. For growth conditions in nutrient-poor medium, the phosphate–carbon–nitrogen (PCN) minimal medium buffered with 80 mM MES [2-(N-morpholino) ethanesulfonic acid] was used. The composition of PCN medium is: 4 mM Tricine [N-[tris(hydroxymethyl) methyl]glycine], 0.1 mM FeCl_3_, 376 µM K_2_SO_4_, 150 mM NaCl, 15 mM NH_4_Cl, 1 mM MgSO_4_, 1 µM CaCl_2_, 0.4% (w/v) glucose, 0.4 mM inorganic phosphate (P_i_), and micronutrients (56). The 80 mM MES solution used to buffer the medium was adjusted to the desired pH value with NaOH. To grow strains bearing genetic elements conferring antibiotic resistance, media were supplemented with chloramphenicol (10 µg/mL), kanamycin (30 or 60  µg/mL, in neutral or acid pH, respectively), or ampicillin (100  µg/mL).

**TABLE 1:** *S.* Typhimurium strains used in this study^*a*^

Strain	Relevant genotype (*)	Source/ reference
SV5015	SL1344 *hisG*^+^	(55)
MD5052	*∆mrdA* (∆*PBP2*)	(26)
MD2576	∆*PBP2*_*SAL*_	(26)
MD4356	∆*ftsI* (∆*PBP3*)	(25)
MD2577	∆*PBP3*_*SAL*_	(25)
MD5064	*PBP2*_*SAL*_-3xFLAG *PBP3*_*SAL*_-3xFLAG	(26)
MD5516	∆*mrdA* PBP2_*SAL*_-3xFLAG *PBP3*_*SAL*_-3xFLAG::Km^R^	(31)
MD5098	∆*PBP2*_*SAL*_ *PBP3*_*SAL*_-3xFLAG::Km^R^	(31)
MD5534	∆*PBP3 PBP2*_*SAL*_-3xFLAG *PBP3*_*SAL*_-3xFLAG::Km^R^	This work
MD5099	∆*PBP3*_*SAL*_ *PBP2*_*SAL*_-3xFLAG::Km^R^	This work
MD1158	SL1344 *sseJ*-3xFLAG::Km^R^	This work
MD1189	*ssrA*::*Mud*J *sseJ*-3xFLAG	This work
MD5068	∆*mrdA* (∆*PBP2*) *sseJ*-3xFLAG	This work
MD5065	∆*PBP2*_*SAL*_ *sseJ*-3xFLAG	This work
MD5069	∆*PBP3 sseJ*-3xFLAG	This work
MD5066	∆*PBP3*_*SAL*_ *sseJ*-3xFLAG	This work
MD0170	SL1344 *prgK*::*aphT* (Km^R^)	(23)
MD0706	SL1344 ∆SPI-1::Km^R^	(33)
MD3369	∆SPI-1::Km^R^	This work
MD2591	∆*mrcA* (∆*PBP1A*)	(31)
MD2569	∆*mrcB* (∆*PBP1B*)	(31)
JPTM7	SL1344 *hilA*-3xFLAG::Km^R^	(57)
MD5712	∆*mrdA* (∆*PBP2*) *hilA*-3xFLAG::Km^R^	This work
MD5713	∆*PBP2*_*SAL*_ *hilA*-3xFLAG::Km^R^	This work
SV5934	*hilC*-3xFLAG	J. Casadesús
MD5714	∆*mrdA* (∆*PBP2*) *hilC*-3xFLAG::Km^R^	This work
MD5715	∆*PBP2*_*SAL*_ *hilC*-3xFLAG::Km^R^	This work
SV5624	14028 *hilD*-HA	(58)
MD5722	*hilD*-HA	This work
MD5723	∆*mrdA* (∆*PBP2*) *hilD*-HA	This work
MD5724	∆*PBP2*_*SAL*_ *hilD*-HA	This work
SV5457	14028 *invF*-3xFLAG::Km^R^	(58)
MD5719	*invF*-3xFLAG::Km^R^	This work
MD5720	∆*mrdA* (∆*PBP2*) *invF*-3xFLAG::Km^R^	This work
MD5721	∆*PBP2*_*SAL*_ *invF*-3xFLAG::Km^R^	This work

^*a*^ (*) Except indicated, all listed strains are isogenic to SV5015.

### Phage transductions

The phage P22 HT 105/1 *int*201 (59) was used for transduction crosses to mobilize chromosomal HA- or 3 × FLAG-epitope-tagged genes alleles for strain construction.

### Bacterial infection of macrophage and fibroblasts

The murine macrophage line RAW 264.7 (ATCC TIB-71) was propagated at 37°C in a 5% CO_2_ atmosphere in Dulbecco’s modified Eagle’s medium (DMEM) with 5% (v/v) fetal bovine serum (FBS) and 4 mM L-glutamine. For infection, RAW 264.7 macrophages were cultured in 24-well tissue culture plates and incubated before infection for 24 h in DMEM-5% FBS medium containing 2.5 ng/mL interferon-γ. The culture was replaced by fresh medium without interferon-γ and the RAW 264.7 macrophages exposed for 10 min to the *S*. Typhimurium isogenic strains lacking morphogenetic PBPs (Table 1) at a multiplicity of infection (MOI) of 10:1. In all cases, bacteria were grown overnight in non-shaking conditions at 37°C in LB pH 5.8 medium. The infected macrophages were washed with phosphate-buffered saline (PBS) supplemented with 0.5 mM MgCl_2_ and 0.9 mM CaCl_2_ and fresh DMEM–5% FBS medium containing gentamicin (100 µg/mL for 1 h, followed by 10 µg/mL for the remaining incubation) to kill extracellular non-phagocytosed bacteria. Number of viable intracellular bacteria was calculated after plating serial dilutions of extracts obtained by lysing the macrophages in a 1% Triton–X100 PBS solution, as described (25). For non-phagocytic cells, the NRK-49F (ATCC CRL-1570) rat fibroblast infection model, was used (60, 61). NRK-49F fibroblasts were propagated in DMEM–5% FBS medium containing 2 mM L-glutamine and cultured in 24-well plates at 50%–60% confluency the day before infection. Fibroblasts were exposed to *S*. Typhimurium morphogenetic mutants added at a MOI 10:1 (bacteria:fibroblast) and co-incubated for 20 min, time at which medium was replaced by fresh new medium containing 100 µg/mL gentamicin to kill non-internalized extracellular bacteria. The concentration of the antibiotic was lowered at 10 µg/mL at 2 h post-infection for those samples processed at late times (24 h). Similar to macrophages, viable intracellular bacteria were quantified after plating serial dilutions of extracts obtained by lysing the infected fibroblasts in a 1% Triton–X100 PBS pH 7.4 solution, as previously described (61).

### Protein extracts and Western blotting

Bacteria were grown in different conditions to stimulate either SPI-1 or SPI-2 expression. For SPI-1, bacteria were grown for 18 h in LB pH 5.8 medium at 37°C without shaking. For SPI-2, bacteria were first grown overnight in the nutrient-limited PCN pH 4.6 150 mM NaCl medium supplemented with 0.2% (w/v) casamino acids at 37°C with shaking. This culture was diluted in the next day in PCN pH 4.6 150 mM NaCl medium with 0.4% (w/v) glucose to initial OD_600_ values of ~0.025. Bacteria with induced SPI-2 expression were collected when reaching OD_600_ ~0.15–0.20. Protein extracts containing cell-associated proteins or proteins secreted to the culture supernatant were prepared essentially as described (23). Briefly, bacteria were spun down by centrifugation (15,500×*g*, 15 min, 4°C), and the supernatant was filtered through 0.45 µm filter (Millipore) to remove residual bacteria. Proteins present in this supernatant were precipitated in 10% (w/v) trichloroacetic acid on ice overnight. Precipitated proteins were collected by centrifugation (30,000*×g*, 45 min, 4°C), washed in ice-cold acetone and allowed to dry. Samples were resuspended in 40 µL of Laemmli buffer. For cell-associated proteins, the bacterial pellets were resuspended in an appropriate volume (100–150 µL) of cold PBS pH 7.4 and a corresponding volume of concentrated fourfold Laemmli buffer. Proteins were resolved by SDS-PAGE and the samples processed for Western blotting, as described (31). The following primary antibodies were used: mouse monoclonal anti-HA (BioLegend, catalogue no. 901533, dilution: 1:2,000) and anti-FLAG (Merck/Sigma-Aldrich, catalogue no. F3165, dilution 1:5,000) antibodies; affinity-purified rabbit polyclonal anti-PBP3 (1:1,000; our laboratory collection); affinity-purified rabbit polyclonal anti-PBP2 (1:500, our laboratory collection); rabbit polyclonal anti-PrgK (gift of Dr. S.I. Miller, University of Washington, USA); rabbit polyclonal anti-SipC (our laboratory collection); and rabbit polyclonal rabbit anti-SseC (gift of Dr. M. Hensel, Osnabrüeck University, Germany). As secondary antibodies, goat polyclonal anti-mouse-HRP (Bio-Rad, catalog no. 115-035-020, dilution 1:10,000) and goat anti-rabbit IgG-HRP (Bio-Rad, catalog no. 111-035-003, dilution 1:25,000) were used. A ChemiDoc Imaging Systems (Bio-Rad) was used for antibody-mediated detection. Signal intensities of the specific bands in the Western blot and two major bands in the Coomassie-stained polyvinylidene difluoride (PVDF) membrane, were determined by densitometry using ImageJ2 version 2.14.0/1.54 f (https://imagej.net/ij/).

### Microscopy

At the desired growth conditions, bacteria were fixed with 3% (w/v) paraformaldehyde (PFA) for 20 min at RT and adjusted to a final 1% PFA concentration. The fixed bacteria were centrifuged (4,300*×g*, 2 min, RT) and resuspended in PBS buffer pH 7.4. A volume corresponding to 50  µL of bacterial suspension was dropped on poly-L-lysine-precoated coverslips and incubated for 15 min at RT. Bacteria attached to the poly-L-lysine-coated coverslip were washed three times with PBS, and the coverslip was mounted on slides using ProLong Gold Antifade (Molecular Probes). Images were acquired on an inverted Leica DMI 6000B microscope with an automated CTR/7000 HS controller (Leica Microsystems) and an Orca-R2 charge-coupled-device (CCD) camera (Hamamatsu Photonics).

### Motility assay

To monitor the motility of *S*. Typhimurium morphogenetic mutants, bacteria were incubated overnight at 37°C in liquid motility medium (1% tryptone and 1% NaCl) adjusted to pH 4.6 with 80 mM MES and in shaking conditions to reach stationary phase. These bacteria were inoculated onto plates containing semi-solid motility medium (1% tryptone, 1% NaCl, 0.25% agar) pH 4.6 by puncturing with a sterile tip previously introduced into the liquid culture inoculum. The plates were incubated at 37°C for approximately 6 h.

### Statistical analyses

Data were analyzed by one-way ANOVA with multiple comparison and Tukey’s post-test. Statistical significance was established at *P* ≤ 0.05.
